# Assessment of CcpA-mediated catabolite control of gene expression in *Bacillus cereus *ATCC 14579

**DOI:** 10.1186/1471-2180-8-62

**Published:** 2008-04-16

**Authors:** Menno van der Voort, Oscar P Kuipers, Girbe Buist, Willem M de Vos, Tjakko Abee

**Affiliations:** 1 TI Food and Nutrition, Wageningen, The Netherlands; 2 Department of Molecular Genetics, University of Groningen, Haren, The Netherlands; 3 Laboratory of Food Microbiology, Wageningen University, Wageningen, The Netherlands; 4 Department of Medical Microbiology, University Medical Center Groningen and University of Groningen, Groningen, The Netherlands

## Abstract

**Background:**

The catabolite control protein CcpA is a transcriptional regulator conserved in many Gram-positives, controlling the efficiency of glucose metabolism. Here we studied the role of *Bacillus cereus *ATCC 14579 CcpA in regulation of metabolic pathways and expression of enterotoxin genes by comparative transcriptome analysis of the wild-type and a *ccpA*-deletion strain.

**Results:**

Comparative analysis revealed the growth performance and glucose consumption rates to be lower in the *B. cereus *ATCC 14579 *ccpA *deletion strain than in the wild-type. In exponentially grown cells, the expression of glycolytic genes, including a non-phosphorylating glyceraldehyde-3-phosphate dehydrogenase that mediates conversion of D-glyceraldehyde 3-phosphate to 3-phospho-D-glycerate in one single step, was down-regulated and expression of gluconeogenic genes and genes encoding the citric acid cycle was up-regulated in the *B. cereus ccpA *deletion strain. Furthermore, putative CRE-sites, that act as binding sites for CcpA, were identified to be present for these genes. These results indicate CcpA to be involved in the regulation of glucose metabolism, thereby optimizing the efficiency of glucose catabolism. Other genes of which the expression was affected by *ccpA *deletion and for which putative CRE-sites could be identified, included genes with an annotated function in the catabolism of ribose, histidine and possibly fucose/arabinose and aspartate. Notably, expression of the operons encoding non-hemolytic enterotoxin (Nhe) and hemolytic enterotoxin (Hbl) was affected by *ccpA *deletion, and putative CRE-sites were identified, which suggests catabolite repression of the enterotoxin operons to be CcpA-dependent.

**Conclusion:**

The catabolite control protein CcpA in *B. cereus *ATCC 14579 is involved in optimizing the catabolism of glucose with concomitant repression of gluconeogenesis and alternative metabolic pathways. Furthermore, the results point to metabolic control of enterotoxin gene expression and suggest that CcpA-mediated glucose sensing provides an additional mode of control in moderating the expression of the *nhe *and *hbl *operons in *B. cereus *ATCC 14579.

## Background

*Bacillus cereus *is an important Gram-positive, spore-forming food-borne pathogen. Many strains cause either an emetic or a diarrhoeal type of disease. The production of emetic toxin in foods, also referred to as cereulide, may cause nausea and vomiting. The diarrhoeal type of disease is associated with the production of enterotoxins in the intestines and may involve Nhe, Hbl and CytK [1-3]. Food-borne disease caused by *B. cereus *is generally characterized by mild symptoms. However, recently more severe cases with a lethal outcome have been described [4,5]. *B. cereus *can also be the causative agent of other diseases, such as periodontitis, fulminant endophthalmitis, and meningitis in immuno-compromised patients [1,6-8]. *B. cereus *is ubiquitously found in the environment, including in soil. Therefore, the transfer to food is not surprising and causes many problems [1]. In nutrient-rich environments, such as food, *B. cereus *shows low generation times putatively gaining advantage from its capacity to use various carbohydrates and proteinaceous substrates [9]. The regulation of gene expression plays an important role in the efficient selection of the preferred carbon and energy source for growth. Annotation of the genome of *B. cereus *ATCC 14579 predicted the regulation of gene expression to be highly complex involving over two hundred transcriptional regulators managing its 5370 open reading frames (ORFs) [9,10]. One of these putative regulators is the catabolite control protein CcpA, which is a member of the LacI-family of transcriptional regulators. CcpA and the regulatory mechanism of the catabolite repression are highly conserved in low-GC Gram-positives [11]. *B. cereus *ATCC 14579 CcpA shows 77% identity with *B. subtilis *CcpA. Furthermore, CcpA in *B. subtilis *has been shown to have a role in optimizing glucose metabolism and the underlying regulatory mechanisms have recently been reviewed [12-14]. Regulation of gene expression by CcpA is mediated by its binding to DNA at a specific cis-binding sequence, the Catabolite Responsive Element (CRE) [14-16].

In recent years the regulon of *B. subtilis *CcpA has been studied extensively by transcriptome analyses, revealing genes and operons under direct and indirect control of CcpA [17-20]. Furthermore, Moreno *et al. *[21] showed a clear correlation between the glucose-repressed genes and the presence of predicted CRE-sites. Moreover, they showed CcpA-mediated glucose-independent regulation of expression [21]. Other organisms for which the role of CcpA in carbon metabolism was established are *Lactobacillus acidophilus *[22] and *Lactococcus lactis *[23]. Recently, a role for CcpA in the control of virulence of *Staphylococcus aureus *[24], *Streptococcus pneumoniae *[25], and *Clostridium perfringens *was reported [26] and reviewed [27].

Notably, comparative genomics of the different species of the *B. cereus *group revealed reduced capacity to metabolize carbohydrates and increased potential for protein metabolism as compared to *B. subtilis *[28,29]. Here we report on the role of CcpA in regulation of metabolism and virulence in *B. cereus *ATCC 14579.

## Results and Discussion

### Growth and glucose utilization of the *ccpA *deletion strain compared to the wild-type

**Figure 1:**
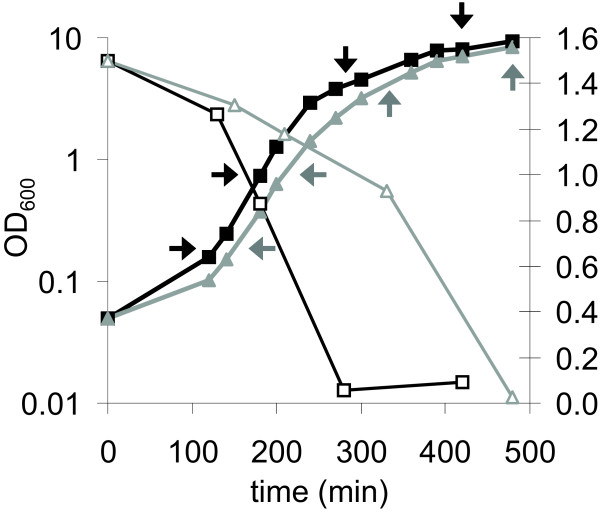
**Growth and glucose consumption**. Growth (closed symbols) and glucose consumption (open symbols) of the wild-type (black) and the *ccpA *deletion strain (grey) in BHI. Arrows in indicate sampling points at OD_600 _of 0.2 (early-exponential), 0.8 (mid-exponential), 4.0 (transition) and 8.0 (stationary), for both glucose determination and transcriptional analysis. Experiments shown are representative for all performed experiments.

Growth of the wild-type and the *ccpA *deletion strain was assessed under aerobic conditions in BHI containing 2 g/L D-glucose, and revealed specific growth rates (μ) of 0.024 and 0.022 (h^-1^), respectively (Fig. 1). Statistical analysis showed the growth rates of the wild-type and deletion strain to be significantly different. Assessment of glucose concentrations at different time points during growth revealed a reduction in the glucose consumption rate for the *ccpA *deletion strain compared to that of the wild-type (Fig. 1). The glucose concentration at early-exponential growth for both the wild-type and the *ccpA *deletion strain was around 1.2 g/L, and glucose was still available in the mid-exponential growth phase. Notably, glucose was depleted in the wild-type culture upon entry into the transition phase, whereas glucose depletion in the *ccpA *deletion strain culture was only observed in the stationary phase of growth.

### Overview of the transcriptome data

**Figure 2:**
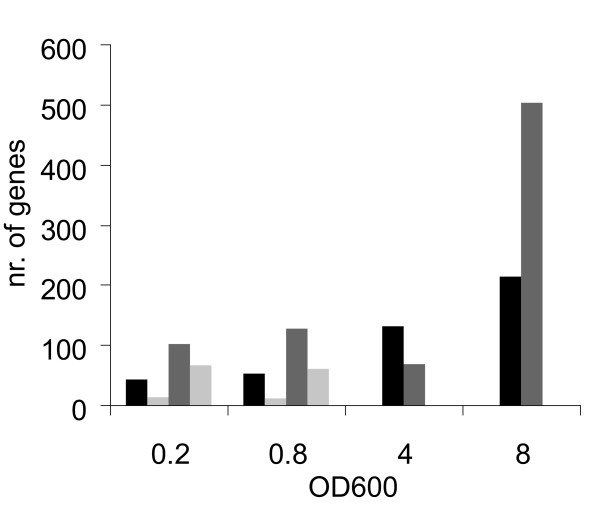
**Differential gene expression during growth**. The number of genes differently expressed in the *ccpA *deletion strain compared to the wild-type in four different growth phases (OD_600 _0.2 = early-exponential, OD_600 _0.8 = mid-exponential, OD_600 _4 = transition, OD_600 _8 = stationary). Both, the genes with a higher (grey bars) and a lower (black bars) expression in the *ccpA *deletion strain are shown. The light grey bars indicate the number of genes putatively regulated by a CRE-site, these are not shown for the transition and stationary phase.

Analyses of the transcriptome data of the *ccpA *deletion strain compared to the wild-type in samples taken at the four time points indicated in figure 1, showed expression of a large number of ORFs to be affected upon *ccpA *deletion. Remarkably, the number of genes differentially expressed increased from 147 at early exponential phase to over 700 genes in the stationary phase (Fig. 2). The large differences in gene expression in transition and stationary phase cells of the *ccpA *deletion strain compared to that of the wild-type are conceivably affected by the respective presence and absence of glucose. Furthermore, these differences may point to the initiation of secondary effects of the *ccpA *deletion in these growth phases on gene expression and consequently cellular performance. Therefore, transcriptome analysis was focused on the early- and mid-exponential phase samples where glucose is still present at high levels in the cultures of both the *ccpA *deletion strain and the wild-type. In early-exponential phase, 103 genes showed higher expression and 44 genes showed a lower expression in the *ccpA *deletion strain compared to the wild-type. For mid-exponential phase cells these numbers were 127 and 54, respectively. When corrected for overlap between regulated genes in the early- and mid-exponential phase, a total of 173 genes expression in the exponential phase was shown to be higher in the *ccpA *deletion strain compared to the wild-type and for 80 genes expression in the *ccpA *deletion strain was observed to be lower than in the wild-type (Fig. 2). Consequently, genes that show a higher expression in the *ccpA *deletion strain are possibly repressed by CcpA, whereas genes that show a lower expression in the *ccpA *deletion strain may be activated by CcpA in the wild-type. The number of genes putatively regulated are similar to those described for CcpA-regulated genes in *B. subtilis *[17-19,21].

Expression ratios of six randomly chosen genes with significantly altered expression in the *ccpA *deletion strain (*cggR*, *acoR*, *gapB*, *ymfC*, *fruR *and *odhA*) were quantified using qPCR. Expression ratios obtained by microarray analysis were compared to ratios obtained by qPCR. The tested genes showed the same trend in expression, with differential expression only slightly more pronounced in qPCR experiments, a feature observed before in such comparisons [30]. This indicates the microarray platform to be suited for gene expression analysis (for qPCR data see Additional file 1).

### Identification of the *B. cereus *CRE-site consensus and *in silico *analysis

**Figure 3:**
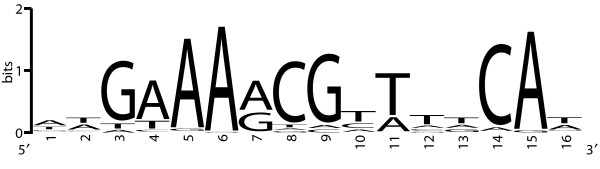
**Visualization of the *B. cereus *ATCC 14579 CRE-site consensus**. The consensus sequence was obtained by aligning putative CRE-sites identified on the genome of *B. cereus *ATCC 14579 in or in front of differently expressed genes between the *ccpA *deletion strain and the wild-type.

Direct regulation exerted by CcpA is indicated by the presence of a CRE-site. By a series of subsequent alignments searches, the *B. cereus *ATCC 14579 consensus CRE-site was identified and visualized in Figure 3 (for details see Methods). This showed the consensus sequence to consist of only 16-bp for *B. cereus *ATCC 14579, WWGWAARCGWWWWCAW, whereas an 18-bp consensus sequence was reported for *B. subtilis*, WWTGNAARCGNWWWCAWW, [15]. Nevertheless, a high similarity between these consensus sequences exists. The *B. cereus *ATCC 14579 genome was scanned with the obtained CRE-site consensus (Fig. 3), identifying the putative CRE-sites (see Additional file 2). A number of 76 putative CRE-sites were identified for 83 out of 173 genes that show a higher expression in the *ccpA *deletion strain compared to the wild-type in exponential phase cells, and these 83 genes are part of 49 putative operons [9,31]. Furthermore, 21 putative CRE-sites were identified in the promoter region of 18 out of 80 genes with a lower expression in the *ccpA *deletion strain (see Additional file 2) in exponential phase cells, these 18 genes are part of 17 putative operons [9,31]. These results are in agreement with the general observation that activation by CcpA occurs less frequently than repression by CcpA [21]. For *B. subtilis *not all identified putative CRE-sites could be shown to be functional [15] and the predicted CRE-sites in *B. cereus *should therefore be regarded putative.

### Analysis of genes differentially expressed in the *ccpA *deletion strain

**Figure 4:**
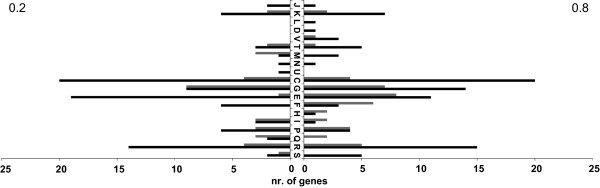
**Functional classes of differentially expressed genes**. Functional classes according to COG to which the differently expressed genes in the *ccpA *deletion strain compared to the wild-type belong, for both the early-exponential (0.2) and mid-exponential (0.8) growth phase. Both, genes with a higher (black) and a lower (grey) expression in the *ccpA *deletion strain are shown. COG categories indicated are J: Translation, K: Transcription, L: Replication, recombination and repair, D: Cell cycle control, mitosis and meiosis, V: Defense mechanisms, T: Signal transduction mechanism, M: Cell wall/membrane biogenesis, N: Cell motility, U, Intracellular trafficking and secretion, C: Energy production and conversion, G: Carbohydrate TM, E: Amino acid TM, F: Nucleotide TM, H: Coenzyme TM, I: Lipid TM, P: Inorganic ion TM, Q: Secondary metabolites TM, R: General function prediction only, S: Function unknown. TM: Transport and metabolism.

The COG annotation reveals that the differentially expressed genes are mainly involved in metabolic processes (Fig. 4), including glycolytic and gluconeogenic genes and genes encoding the citric acid cycle (Table 1). For approximately 35 differentially expressed genes a homologous gene was identified to be CcpA regulated in *B. subtilis *[32]. Of these genes a total number of 17 genes were annotated to have a function in the glucose metabolism (Table 1). Genes that were first identified in this study to be putatively regulated by CcpA with an apparent and interesting function are discussed below.

**Table 1:** Genes involved in *B. cereus *ATCC14579 glucose metabolism and their putative CRE-sites

**Gene**	**Operon**^¶^	**Annotation**	**BC nr.**	**RZC nr.**	**Δ*ccpA*/WT †**		**CRE‡**
					**early**	**mid**	

**Glycolysis**							

ptsG^#^	1	PTS system, glucose-specific IIABC component	BC4050	RZC06596	0.52	1.12	2
ptsH^#^	2	Phosphocarrier protein HPr	BC4049	RZC00081	0.56	1.10	+
ptsI^#^	3	Phosphoenolpyruvate-protein phosphotransferase	BC4048	RZC06595	0.66	1.16	1
pgi		Glucose-6-phosphate isomerase	BC4898	RZC03793	0.31	0.20	1
pfkA	1	6-phosphofructokinase	BC4600	RZC02283	0.45	0.47	-
pykA	2	Pyruvate kinase	BC4599	RZC01414	0.29	0.33	-
fbaA		Fructose-bisphosphate aldolase	BC5335	RZC03557	0.39	0.52	2
cggR^#^	1	Central glycolytic genes regulator	BC5141	RZC08032	0.49	0.73	-
gapA^#^	2	Glyceraldehyde-3-phosphate dehydrogenase	BC5140	RZC00210	0.36	0.59	-
pgk* ^#^	3	Phosphoglycerate kinase	BC5139	RZC00211	0.49	0.41	-
pgk* ^#^	3	Phosphoglycerate kinase	BC5138	RZC08031	0.53	0.46	-
tpiA^#^	4	Triose-phosphate isomerase	BC5137	RZC08030	0.62	0.51	-
pgmA^#^	5	Phosphoglycerate mutase	BC5136	RZC05843	0.48	0.38	-
eno^#^	6	Phosphopyruvate hydratase	BC5135	RZC02971	0.42	0.51	-
gapN		NADP-dependent glyceraldehyde-3-P dehydrogenase	BC0868	RZC03841	0.31	0.26	2

**Pyruvate dehydrogenase**							

pdhA^#^	1	Pyruvate dehydrogenase (acetyl-transferring)	BC3973	RZC01920	1.24	0.77	2
pdhB^#^	2	Pyruvate dehydrogenase (acetyl-transferring)	BC3972	RZC01921	1.47	0.79	+
pdhC^#^	3	Dihydrolipoyllysine-residue acetyltransferase	BC3971	RZC06754	1.52	0.95	+
pdhD^#^	4	Dihydrolipoyl dehydrogenase	BC3970	RZC07919	1.28	1.01	+

**Overflow metabolism**							

alsS^§^	1	Acetolactate synthase large subunit	BC0883	RZC01898	1.04	0.54	2
alsD^§^	2	Alpha-acetolactate decarboxylase	BC0884	RZC01896	0.92	0.58	+
Ldh		L-lactate dehydrogenase	BC1924	RZC05932	0.44	1.05	-
lctP		L-lactate permease	BC0612	RZC01585	0.34	1.21	-
pta^§^		Phosphate acetyltransferase	BC5387	RZC05214	0.61	0.70	1
ackA^§^		Acetate kinase	BC4637	RZC02436	0.93	1.29	1

**Citric acid cycle**							

citZ^§^	1	Citrate synthase	BC4594	RZC01265	3.76	2.23	1
citC^§^	2	Isocitrate dehydrogenase [NADP]	BC4593	RZC01263	5.56	4.38	1
mdh^§^	3	Malate dehydrogenase	BC4592	RZC01264	5.81	4.32	+
citB^§^		Aconitate hydratase	BC3616	RZC04993	1.62	1.26	1
odhA^§^	1	Oxoglutarate dehydrogenase	BC1252	RZC06639	2.07	3.80	4
odhB^§^	2	Dihydrolipoyllysine-residue succinyltransferase	BC1251	RZC03695	2.36	3.97	+
sucC^§^	1	Succinate-CoA ligase (ADP-forming)	BC3834	RZC02282	8.23	6.32	1
sucD^§^	2	Succinate-CoA ligase (ADP-forming)	BC3833	RZC02281	7.74	7.57	+
sdhC^§^	1	Succinate dehydrogenase	BC4518	RZC05750	4.02	4.02	1
sdhA^§^	2	Succinate dehydrogenase	BC4517	RZC07054	3.70	3.70	1
sdhB^§^	3	Succinate dehydrogenase	BC4516	RZC07053	3.76	5.64	+
citG^§^		Fumarate hydratase	BC1712	RZC03022	1.29	1.49	2

**Gluconeogenensis**							

fbpA		Fructose-bisphosphatase	BC4962	RZC03080	0.75	0.74	2
gapB		Glyceraldehyde-3-P dehydrogenase	BC4583	RZC07229	1.74	2.79	3
pckA		Phosphoenolpyruvate carboxykinase (ATP)	BC4762	RZC07251	1.37	1.09	1
ywjI		Fructose-bisphosphatase	BC5333	RZC00125	3.73	3.34	+

§ Genes of which expression of their homologous gene in *B. subtilis *was CcpA dependent [18,19,21].

# Genes of which effects of *ccpA *deletion on expression were shown to be indirect in *B. subtilis *[18, 19, 21].

* pgk is represented on the microarray by two different amplicons, as Pgk is annotated in the ERGO database to be encoded by two genes.

¶ Numbers indicate the order of genes within one operon.

† Ratios are presented as expression in the *ccpA *deletion strain compared to the wild-type (WT), expression in the wild type is set to 1. Early- and mid-exponential phase data are shown.

‡ In this column it is indicated whether a putative CRE-site was identified for the gene. Numbers indicate the number of putative CRE-sites identified, a + indicates that a gene is in the same operon as a gene for which a putative CRE-site was identified, and a - indicates no putative CRE-site could be identified.

One of the glycolytic genes found to be expressed lower in the *ccpA *deletion strain was *yfmT*. This gene encodes a non-phosphorylating glyceraldehyde-3-phosphate dehydrogenase (GAPN), for which 2 putative CRE-sites could be identified. We propose to rename *yfmT *as *gapN*, since this gene shows similarity with *gapN *of the other members of the *B. cereus *group, and with *gapN *of *B. halodurans*, *Streptococci *and *Clostridia *[33]. Notably, the gene is lacking in *B. subtilis *[29,33]. Next to the non-phosphorylating glyceraldehyde-3-phosphate dehydrogenase, two phosphorylating glyceraldehyde-3-phosphate dehydrogenases encoding genes (*gapA *and *gapB*) are present on the *B. cereus *ATCC 14579 genome. The function of GAPN in microbial metabolism is yet unclear. Recently, Asanuma and Hino (2006) showed a role for CcpA in expression control of *gapN *in *Streptococcus bovis *and proposed that NADPH is provided by GAPN activity for NADPH-dependent biosynthetic reactions, thereby maintaining an optimal redox balance at the same time. However, in hyperthermophilic archaea GAPN has been shown to play a role in accelerating glycolysis as it can produce 3-phospho-D-glycerate from D-glyceraldehyde 3-phosphate in a one-step reaction, instead of two steps[34]. As a putative drawback, ATP is not produced in this one-step reaction. The exact role of GAPN in *B. cereus *metabolism remains to be elucidated.

The gluconeogenic genes expressed higher in the *ccpA *deletion strain included *ywjI *and *gapB*. The *ywjI *gene is part of the putative *murA2*-*ywjI *operon and is annotated as a fructose-1,6-bisphosphatase. The *ywjI *gene of *B. subtilis*, that shows similarity to *ywjI *of *B. cereus*, is annotated to be a fructose-1,6-bisphosphatase, as a member of the *glpX*-family. The other annotated fructose-1,6-bisphosphatase gene for *B. subtilis*, *fbp*, which is no member of the *glpX*-family, is not regulated by CcpA and deletion of this gene seems to have no effect on gluconeogenesis [35]. A corresponding *fbp *homologue appears to be absent in *B. cereus*. However, on the *B. cereus *genome two members of the *glpX*-family of fructose-1,6-bisphosphatases are present, of which *ywjI *expression was found to be affected by *ccpA *deletion, accordingly a putative CRE-site was identified in the promoter region of its operon. The *gapB *gene encodes a NAD(P)-dependent glyceraldehyde-3-phosphate dehydrogenase. Three putative CRE-sites were identified for *gapB *in *B. cereus *(Table 1). In contrast, no CRE-sites were identified for *gapB *in *B. subtilis*, and its expression was shown to be only indirectly affected by *ccpA *deletion [36]. Furthermore, gluconeogenesis in *B. subtilis *was shown to be regulated by the transcriptional regulator CcpN [37,38]. CcpN is also present on the genome of *B. cereus *ATCC 14579 and putative CcpN binding sites can be found in front of the gluconeogenic genes encoding GapB and PckA [37]. Combined with our results this would point to dual control of the expression of gluconeogenic genes in *B. cereus *by CcpN and CcpA.

**Figure 5:**
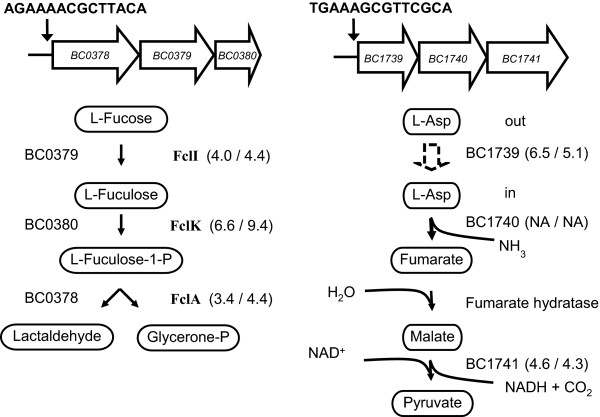
**Putative CcpA-controlled metabolic pathways in *B. cereus***. Two operons observed to be higher expressed in the *ccpA *deletion strain compared to the wild-type with their proposed functions and between brackets the expression in the ccpA deletion strain compared to the wild-type in the early- and mid-exponential growth phase, respectively. Putative CRE-sites are indicated in front of the operon, the open arrow in the flow scheme stands for transport into the cell, whereas the closed arrows stand for reactions. L-Asp stands for L-Aspartate and P stands for phosphate.

The expression of genes with functions in routing metabolism towards the citric acid cycle appeared to be higher in the *ccpA *deletion strain compared to the wild-type and included ~30 genes involved in amino acid catabolism. This included the *bkd*-operon (BC4163 to BC4157) that is common among the *Bacilli*, and that is involved in the catabolism of Valine and Leucine [39]. One putative CRE-site in front of the *bkd*-operon and two within the operon were identified in *B. cereus *ATCC 14579. Notably, CcpA was previously shown to play no role in the regulation of expression of this operon in *B. subtilis *[40]. In contrast, the *ilv-leu*-operon of *B. subtilis *coding for biosynthesis of branched chain amino acids has been shown to be activated by CcpA [41], whereas no involvement of CcpA was found in regulation of expression of this operon in *B. cereus *under the conditions tested. The fact that no regulation of the *ilv-leu*-operon was observed, although its expression on the microarray was apparent (data not shown), could be due to a complex regulation of this operon under the tested circumstances involving CodY and TnrA [41,42]. Expression of several genes encoding enzymes involved in catabolism of nucleosides was higher in the *B. cereus ccpA *deletion strain, this included the putative *yuf*-operon (BC3791 to BC3788) encoding nucleoside transport under the apparent control of the transcriptional regulator encoded by the also higher expressed *ymfC *(BC3792), for both the regulator and the *yuf*-operon putative CRE-sites could be identified (see Additional file 2). Interestingly, expression of two putative operons with an apparent function in catabolism was higher in the *ccpA *deletion strain, and possible functions were assessed using *in silico *analysis. The first operon (BC0378-BC0380), which is not present in *B. subtilis*, consists of three genes and based on phylogenetic studies of the protein sequences it is suggested to annotate the genes as an L-fuculose phosphate aldolase [43] (BC0378), an L-fucose and/or arabinose isomerase (BC0379) and an L-fuculose phosphate aldolase (BC0380). This suggest a function for this operon in the catabolism of fucose and/or arabinose. Consequently, names and functions that we suggest are *fclI *for the L-fucose isomerase (BC0379), *fclK *for the L-fuculokinase (BC0378) and *fclA *for the L-fuculose phosphate aldolase (BC0380) (Fig. 5). A putative CRE-site could be identified in front of this operon. The second operon consists of three genes and *in silico *analysis of this operon (BC1739-1741) suggested the transporter protein (BC1739) to function as a proton/sodium-aspartate symporter [44], as an alternative to *gltT*. Subsequently, aspartate can be converted into ammonia and fumarate by the aspartate ammonia lyase (BC1740), after which fumarate can be metabolized to malate by one of the two fumarate hydratases encoded on the genome of *B. cereus*. The gene BC1741 encodes a malic enzyme, MalS, which is responsible for converting malate into pyruvate. This suggests that the operon encodes enzymes involved in the catabolism of aspartate to pyruvate. This operon is unique for the species of the *B. cereus *group, as the genes are not found as an operon in genomes of other bacteria. A putative CRE-site was identified for the first gene of the operon (Fig. 5). The two-component system (BC1742-BC1743) next to this operon shows high similarity with the two-component system GlnK-GlnL of *B. subtilis*, which is involved in glutamine utilization [45]. Whether BC1742-BC1743 functions as a two-component system, involved in aspartate sensing and triggering expression of enzymes involved in its metabolism, remains to be elucidated.

Glutamate is a product of protein and amino acid catabolism and acts as link between nitrogen and carbon metabolism, mediated by glutamate synthase and glutamate dehydrogenase, as described for *B. subtilis *[46,47]. The *B. subtilis *genome contains a glutamate synthase (*gltAB*) and two glutamate dehydrogenases (*rocG *and *gudB*) of which the GudB dehydrogenase appears to be inactive [47]. Glutamate biosynthesis in *B. subtilis *has recently been shown to be tightly regulated by interaction of RocG and GltC [48], but these genes are not encoded on the *B. cereus *genome. The microbial glutamate synthases generally belong to the NADPH-GltS family of glutamate synthases and are encoded by two genes [49]. In the glutamate synthase reaction L-glutamate is produced from L-glutamine and 2-oxoglutarate, with the latter compound derived from the citric acid cycle [49]. The *gltAB *operon of *B. subtilis *is suggested to be regulated by CcpA [46,50]. On the genome of *B. cereus *ATCC 14579 only one glutamate synthase gene (*gltA*) is present and its expression at various phases of growth is similar in the wild-type and its *ccpA *deletion strain, indicating that its expression is not regulated by CcpA. Moreover, it is unclear to which family of glutamate synthases GltA of *B. cereus *belongs. Interestingly, *gudB *was the only gene present on the genome of *B. cereus *encoding a glutamate dehydrogenase, and its expression was observed to be clearly higher in the *ccpA *deletion strain (Table 1). Glutamate dehydrogenase is responsible for the reaction from L-glutamate to 2-oxoglutarate linking L-glutamate to the citric acid cycle [51]. Higher expression of *gudB *in the *ccpA *deletion strain compared to the wild-type, together with the fact that it is the only glutamate dehydrogenase annotated on the genome of *B. cereus *suggests that GudB is the active glutamate dehydrogenase for *B. cereus*. This is supported by the fact that the 9-bp sequence, encoding the 3 amino acids causing the inactivity of GudB in *B. subtilis *[47], are absent in the *gudB *sequence of *B. cereus *ATCC 14579. Two putative CRE-sites in front of and one within *gudB *were identified pointing to CcpA-controlled expression in *B. cereus*.

*B. subtilis *contains a large number of carbohydrate catabolic pathways [29], whereas the number of these pathways is limited in *B. cereus*. The observed deficiency of the *B. subtilis ccpA *deletion strain in growth with ammonium as the sole nitrogen-source, has been attributed to the regulation of the *gltAB *genes by CcpA [46,50] and the read-through transcription of the *rocG *gene [47]. The lack of regulation of *gltA *in *B. cereus *by CcpA, together with a similarity to the *B. subtilis gltA *of only 46%, offers an explanation for the observation that the *ccpA *deletion strain of *B. cereus *is able to grow with ammonium as the sole nitrogen source (data not shown), conceivably with GudB as the active glutamate dehydrogenase.

The role of CcpA in optimisation of glucose metabolism is apparent, since glycolytic enzymes were expressed lower and expression of genes encoding citric acid cycle enzymes was shown to be higher in the *ccpA *deletion strain compared to the wild-type. Up-regulation in the *ccpA *deletion strain was found for ~30 genes encoding enzymes involved in protein, peptide and amino acid metabolism (see Additional file 1). This is seemingly in contrast with the proposed preferred use of proteinaceous substrates for growth of *B. cereus*, a hypothesis put forward by Ivanova *et al. *[9]. This hypothesis was supported by the annotation of a large number of genes encoding proteolytic enzymes, a multiplicity of peptide and amino acid transporters, and a large variety of amino acid degradation pathways. However, under the conditions tested glucose is used as the carbon and energy source (Fig. 1) and genes encoding enzymes involved in protein, peptide and amino acid catabolism appear to be subject to catabolite repression, indicating that under the conditions tested, glycolysis rather than protein catabolism is the preferred energy generation route of *B. cereus *ATCC 14579.

### CcpA-mediated catabolite control of enterotoxin gene expression in *B. cereus*

**Figure 6:**
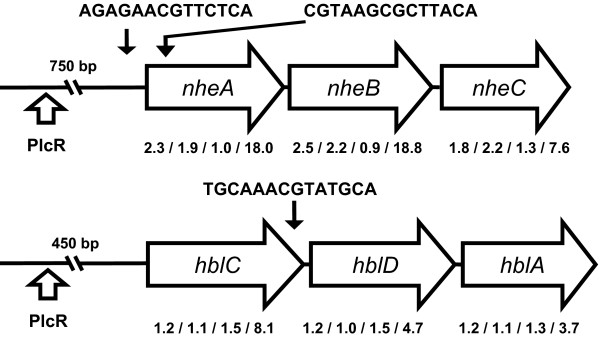
**Putative CcpA-regulated enterotoxin operons**. Positioning of CRE-sites and PlcR binding sites in two major enterotoxin operons encoding Nhe and Hbl of *B. cereus *ATCC 14579. Ratios of gene regulation are given as found in the *ccpA *deletion strain compared to the wild-type, for the four different growth phases, early- and mid-exponential, transition and stationary phase, respectively. Closed arrows indicate the approximate position of the identified CRE-site, open arrows indicate binding sites for the pleiotropic regulator PlcR [59].

In early- and mid-exponential phase cells the expression of the *nhe*-operon (BC1809-BC1811), coding for the non-hemolytic toxin Nhe [52], was shown to be higher in the *ccpA *deletion strain compared to the wild-type. This higher expression was even more apparent in the stationary phase where an almost 20-fold stimulation was observed (Fig. 6). Furthermore, a putative CRE-site was identified for this operon. For the *hbl*-operon (BC3104-BC3102), coding for the HBL enterotoxin [53], an 8-fold increase in the *ccpA *deletion strain was seen in the stationary phase and a putative CRE-site could be identified (Fig. 6). Differential expression of *cytK*, encoding the enterotoxin Cytotoxin K [4], was not observed under the conditions tested, and no putative CRE-site could be identified. These results indicate involvement of CcpA in the catabolite control of the expression of at least two major enterotoxins in *B. cereus *ATCC 14579. Many bacteria use glucose as their preferred carbon and energy source as it can be obtained from plant-derived polysaccharides, which can be found in many environments including foods. We hypothesize that glucose can function as a signalling molecule enabling CRE site-mediated, CcpA-dependent repression of gene expression for microorganisms such as *Lactobacillus plantarum *[54] and *B. cereus *that occur in several environments, including soil, plant and the human gastro intestinal (GI) tract. In the GI tract, in the absence of glucose, CcpA-dependent repression is thus lifted, and this allows for induction of pathways involved in catabolism of other substrates present in the GI tract such as fucose, a major degradation product of mucus related fucosylated glycans [55]. Notably, results of this study suggest the putative fucose utilisation operon of *B. cereus *to be repressed by CcpA in the presence of glucose, and this pathway may thus be activated in the GI-tract under glucose limiting conditions. A similar mode of regulation seems applicable for the *nhe *and *hbl *enterotoxin operons, for which the combination of transcriptome data and the identification of putative CRE-sites, suggest these to be CcpA controlled as well. Our results offer an explanation for the observations recently made by Ouhib *et al. *[56], who showed that final enterotoxin levels reached, depended on the carbon and energy sources used for growth, with the lowest levels reached with cells grown on glucose. The anaerobic growth transcriptional regulators FNR and ResD have recently been found to play a role in enterotoxin production also, next to PlcR [57-59], and combined with our data, this points to an elaborate control of enterotoxin production in *B. cereus *at the transcriptional level based on a variety of input signals mediated by a range of transcriptional regulators. Notably, CcpA has been reported also to play a role in toxin formation in *C. perfringens *[26], and in *S. aureus *deletion of *ccpA *was shown to affect the expression of the virulence gene *hla*, encoding α-hemolysin [24].

## Conclusion

*B. cereus *ATCC 14579 catabolite control protein CcpA is involved in optimizing the catabolism of glucose with concomitant repression of a range of metabolic pathways. Furthermore, CcpA-mediated glucose sensing is shown to provide an additional mode of control in moderating the expression of the *nhe*- and *hbl*-operon in this human pathogen.

## Methods

### Bacterial strains, culture media, growth conditions, and genetic methods

*B. cereus *ATCC 14579 and its *ccpA *deletion strain FM1403 were cultured in brain heart infusion broth (BHI, Becton and Dickinson, The Netherlands) medium at 30°C, with shaking at 200 rpm. The growth of the culture was monitored by measurement of the optical density at OD_600_. D-glucose concentrations were measured by use of a D-glucose measuring kit (Boehringer). Growth experiments and glucose measurements were performed in three fold. Plasmid DNAs were purified from *E. coli *with a Qiaprep Spin Miniprep kit (Westburg, Leusden, The Netherlands). Pwo polymerase (Roche Diagnostics, Almere, The Netherlands) was used for PCR generated fragments that were used in cloning and Taq polymerase (Fermentas, Amersfoort, The Netherlands) was used in control PCRs. *E. coli *HB101/pRK24 [60] was used as the donor host in conjugation experiments. The antibiotics used were ampicillin (Sigma, Zwijndrecht, The Netherlands) at a concentration of 50 μg/ml, kanamycin (Sigma) at a concentration of 70 μg/ml, erythromycin (Sigma) at a concentration of 150 μg/ml (for *E. coli*) or 5 μg/ml (for *B. cereus*), spectinomycin (Sigma) at a concentration of 100 μg/ml, and polymyxin B (VWR, Amsterdam, The Netherlands) at a concentration of 50 μg/ml for counter-selection against *E. coli *upon conjugation.

### Construction of *ccpA *deletion strain

To construct a double cross-over deletion strain of *ccpA*, an ~3.5-kb PCR product, comprising *ccpA *and 1-kb flanking regions was obtained by use of forward primer ccpAKOsacIforw (TCgagctcAGATTACGTTGATGTTATTC) and reverse primer ccpAKOxbaIrev (TGtctagaAGAAGAAGAAAAAGAGGAAGAAAT). This PCR product was cloned into pGemT-easy (Promega, Leiden, The Netherlands) according to the manufacturer's protocol resulting in pGemTccpA. Subsequently, an erythromycin-resistance cassette amplified from pUC18ERY [61] with forward primer ErycasFBsrGI (TCtgtacaGTCCGCAAAAGAAAAACG) and reverse primer ErycasRClaI (TCatcgatCATACCTAATAATTTATCTAC) was cloned into pGemTccpA after digesting both with *Bsp*1407I (*Bsr*GI) and *Bsu*15I (*Cla*I) (Fermentas). The insert of the resulting plasmid, comprising the 1-kb flanking regions and the erythromycin-resistance cassette was cloned into the conjugal vector pATΔS28 [62] by digestion of the insert and the vector with *Xba*I and *Sac*I (Fermentas). The resulting plasmid pATΔCcpAery was isolated from DH5α and transformed into *E. coli *HB101/pRK24. The resulting strain was used in a conjugation experiment with *B. cereus *ATCC 14579 following established procedures [63]. Transconjugants were obtained by selection for spectinomycin sensitivity and erythromycin resistance and one was analysed in comparison with the wild-type strain. PCR and Southern Blot analysis confirmed the deletion of *ccpA *by double homologous recombination (data not shown). The *B. cereus ccpA *deletion strain was designated *B. cereus *FM1403.

### RNA isolation

RNA was extracted from both the *ccpA *deletion strain and the wild-type at four time points in the growth curve at OD_600 _of 0.2, 0.8, 4 and 8 which corresponds to early-exponential, mid-exponential, transition and stationary phase of growth from two independent cultures per phase by using RNAwiz (Ambion, Huntingdon, United Kingdom) according to the manufacturers protocol. Residual DNA from the RNA preparations was enzymatically removed by using TURBO DNA-*free *(Ambion). Extracted RNA samples were stored in 70% EtOH, 0.3 M sodium acetate buffer (pH 5.2) at -80°C.

### Microarray construction and transcriptome analysis

Amplicon based DNA-microarrays were constructed for *B. cereus *ATCC 14579 as described for *L. lactis *IL1403 [64,65] with modifications as detailed below. Amplicons were designed on 5199 genes selected from the 5311 annotated genes (ORFs smaller than 80-bp were omitted) on the genome of *B. cereus *ATCC 14579 [9]. To reduce cross-hybridization between probe and target DNA sequences the amplicons had sizes of 70 – 700-bp (depending on gene sizes) and comprised the most unique part of a gene. The amplicons were synthesized by EuroGentec (Seraing, Belgium) in two amplification steps. In the first amplification step, primers were used with a unique tag-sequence for *B. cereus *ATCC 14579 (forward primers were extended with the sequence: 5'-TCGGGCAGCTGCTCC-3'; and reverse primers with the sequence: 5'-TGGCGCCCCTAGATG-3'). Two copies of each amplicon were present per array, resulting in microarrays comprising 10398 spots.

Normalized expression data (Feature Extract, Agilent) for each spot was used in a statistical analysis. The biological replicate experiments were merged with the web-supported VAMPIRE microarray suite, based on a Bayesian frame work. Furthermore, VAMPIRE calculated p-values for individual spots and subsequently used this p-value to identify statistically differentially expressed spots between compared growth conditions by use of a false discovery rate (FDR) of 0.05 as a threshold [66,67]. In addition, only ORFs of which both individual spots passed the FDR based threshold were considered to be putatively differentially regulated. Expression ratios per ORF were established by calculating the average of the log-values of individual spots. This value (R) was then used to calculate the average expression ratio (10^R^) per ORF. Finally, only ORFs that showed a change in expression of at least 2-fold (up/down) were considered to be differentially expressed. Microarray data are submitted to the GEO database with accession number: GSE7843.

To determine gene similarity, homology and gene context NCBI BLAST and the ERGO database were used [10], while KEGG [68] was used for assessment of metabolic functions and pathways. Whether succeeding genes were part of one operon was determined according to operon prediction as performed by [31].

### Definition and identification of *B. cereus *CRE-site

The 350-bp upstream and 150-bp downstream sequences of the translation start of genes identified by the array experiments to be significantly higher expressed in the *ccpA *deletion strain compared to the wild-type strain for early- and mid-exponential growth were analyzed with AlignACE 3.1 [69], which searches the input sequences for stretches of nucleotides which align between the different input sequences. The 13-bp CRE-sites identified by AlignACE were aligned with MUSCLE 3.6 [70] and a Hidden Markov Model (HMM) was constructed with the HMMER package [71]. The HMM was used to search CRE-sites in the complete genome sequence of *B. cereus *ATCC 14579 [9], and not only the 350-bp upstream and 150-bp downstream sequences significantly higher expressed genes in the *ccpA *deletion strain. Subsequently, to analyse whether the *B. cereus *consensus was longer than 13-bp, the obtained sites after the HMM search were extended to 18-bp, as has been the reported length for *B. subtilis *[15]. The resulting extended CRE-sites were again aligned using MUSCLE 3.6 [70]. This alignment was subsequently visualized with WebLogo [72] and a iteration HMM search was performed on the *B. cereus *ATCC 14579 sequence [9] to identify all putative CRE-sites confirming to the new alignment in the *B. cereus *ATCC 14579 genome.

## Authors' contributions

MV performed the experiments and data analysis and drafted the manuscript. OPK, GB, WMV and TA participated in the design of the study, in evaluation of the results and in revision of the manuscript. All authors read and approved the final manuscript.

## Supplementary Material

Additional file 1 Differentially expressed genes in the *ccpA *deletion strain as compared to the wild-type in early- and mid-exponential phase. Click here for file

Additional file 2 Identified putative CRE-sites on the *B. cereus *ATCC 14579 genome. Click here for file
