# Supplementary material for: Assessment of CcpA-mediated catabolite control of gene expression in Bacillus cereus ATCC 14579
Source: BMC Microbiol. 2008 Apr 16;8:62. doi: 10.1186/1471-2180-8-62 (PMC2358912; doi:10.1186/1471-2180-8-62)
Supplement: Additional file 1 — Differentially expressed genes in the ccpA deletion strain as compared to the wild-type in early- and mid-exponential phase. [file 1471-2180-8-62-S1.doc]

Table S1: Differentially expressed genes in the *ccpA* deletion strain as compared to the wild type in early- and mid-exponential phase.

| RZC nr. | BC nr. | Array ratio early-exp*. | Array ratio mid-exp* | qPCR ratio early-exp† | Alias | Annotation | CRE-site‡ |
| --- | --- | --- | --- | --- | --- | --- | --- |
| RZC01285 | BC0197 | 4.10 | 2.91 |  |  | ABC transporter ATP-binding protein | - |
| RZC04643 | BC0311 | 1.43 | 2.51 |  |  | hypothetical protein | - |
| RZC02596 | BC0372 | 3.43 | 2.62 |  |  | hypothetical protein | + |
| RZC03161 | BC0378 | 6.58 | 9.43 |  | fclK | 5-methylthioribose kinase | 1 |
| RZC02455 | BC0379 | 3.98 | 4.44 |  | fclI | Methylthioribose salvage protein | + |
| RZC02456 | BC0380 | 3.38 | 4.44 |  | fclA | L-fuculose phosphate aldolase | + |
| RZC03422 | BC0410 | 1.07 | 2.91 |  |  | Transcription regulator, Crp family | 1 |
| RZC02626 | BC0467 | 1.73 | 2.33 |  | yfjS | Spore coat N-acetylmuramic acid deacetylase | - |
| RZC05979# | BC0593 | 8.18 | 6.88 |  |  | Alanine permease | -§ |
| RZC01588 | BC0632 | 2.04 | 0.92 |  | treA | Trehalose-6-phosphate hydrolase | - |
| RZC06568 | BC0656 | 3.42 | 6.28 |  | glpT | Glycerol-3-phosphate transporter | 2 |
| RZC05942 | BC0661 | 2.24 | 1.24 |  | rbsD | High affinity ribose transport protein rbsD | 1 |
| RZC01106 | BC0662 | 4.71 | 1.82 |  | rbsA | Ribose transport ATP-binding protein rbsA | + |
| RZC03214 | BC0663 | 14.56 | 10.46 |  | rbsC | Ribose transport system permease protein rbsC | 2 |
| RZC03215 | BC0664 | 10.68 | 8.44 |  | rbsB | D-ribose-binding protein | + |
| RZC07872 | BC0754 | 4.01 | 3.69 |  |  | Potassium-transporting ATPase B chain | - |
| RZC05185 | BC0763 | 1.05 | 3.33 |  |  | ABC transporter permease protein | - |
| RZC05182 | BC0768 | 1.37 | 6.42 |  |  | Methylthioribose-binding protein | - |
| RZC03637 | BC0830 | 1.64 | 3.39 |  |  | hypothetical protein | - |
| RZC03633 | BC0834 | 1.28 | 2.79 |  |  | hypothetical protein | - |
| RZC03213 | BC0923 | 9.96 | 7.80 |  |  | hypothetical protein | - |
| RZC05409 | BC0972 | 0.93 | 4.32 |  |  | hypothetical protein | 1 |
| RZC05136 | BC0999 | 2.40 | 1.09 |  |  | hypothetical protein | - |
| RZC05869 | BC1032 | 1.26 | 4.36 |  |  | Transcriptional repressor | - |
| RZC07888 | BC1034 | 1.41 | 3.06 |  | glpF | Glycerol uptake facilitator protein | 3 |
| RZC00019 | BC1036 | 1.53 | 8.04 |  | glpD | Glycerol-3-phosphate dehydrogenase | + |
| RZC06104 | BC1045 | 2.27 | 1.83 |  |  | hypothetical protein | 1 |
| RZC10760 | BC1054 | 0.75 | 15.37 |  |  | hypothetical protein | - |
| RZC02201 | BC1081 | 1.54 | 2.40 |  |  | PlcR-regulated protein PRP2 | - |
| RZC02199 | BC1082 | 2.13 | 2.10 |  |  | Ribosomal-protein-alanine acetyltransferase | 1 |
| RZC02198 | BC1083 | 10.95 | 8.09 |  |  | Transcriptional regulator, LacI family | 2 |
| RZC07901 | BC1113 | 1.32 | 2.23 |  | yhdL | Sigma-M negative effector | - |
| RZC03609# | BC1149 | 2.16 | 1.51 |  | rocD | Ornithine aminotransferase | 1 |
| RZC03622 | BC1181 | 2.07 | 0.50 |  | oppC | Oligopeptide transport system permease protein oppC | 1 |
| RZC03621 | BC1182 | 2.17 | 0.55 |  | oppD | Oligopeptide transport ATP-binding protein oppD | + |
| RZC06646 | BC1185 | 2.47 | 1.38 |  |  | Oligopeptide-binding protein oppA | 2 |
| RZC02368 | BC1224 | 2.16 | 1.18 |  | yjcF | Acetyltransferase | - |
| RZC02369 | BC1225 | 2.92 | 1.45 |  | yjcG | 2'-5' RNA ligase | - |
| RZC02366 | BC1226 | 2.50 | 1.90 |  | yjcH | Acetyl esterase | - |
| RZC03344# | BC1235 | 2.18 | 2.39 |  | trpC | Indole-3-glycerol phosphate synthase | - |
| RZC03342# | BC1237 | 2.39 | 2.24 |  | trpB | Tryptophan synthase beta chain | - |
| RZC03695 | BC1251 | 2.36 | 3.97 |  | odhB | Dihydrolipoamide succinyltransferase component (E2) of 2-oxoglutarate dehydrogenase complex | + |
| RZC06639 | BC1252 | 2.07 | 3.80 | 4.66 | odhA | 2-oxoglutarate dehydrogenase E1 component | 4 |
| RZC02375 | BC1475 | 0.86 | 2.17 |  |  | ResB protein | - |
| RZC02665 | BC1491 | 4.53 | 4.74 |  | gudB | NAD-specific glutamate dehydrogenase | 3 |
| RZC03717 | BC1508 | 1.83 | 2.47 |  | yphF | hypothetical protein | - |
| RZC03714 | BC1509 | 1.87 | 3.48 |  |  | Stage IV sporulation protein A | - |
| RZC03642 | BC1515 | 7.10 | 8.50 |  |  | Nucleoside diphosphate kinase | 1 |
| RZC03979 | BC1560 | 1.34 | 3.76 |  |  | Spore coat protein D | - |
| RZC03976 | BC1562 | 1.51 | 4.07 |  | ypsB | Cell division protein DIVIVA | - |
| RZC01217 | BC1739 | 6.47 | 5.14 |  |  | Proton/sodium-glutamate symport protein | 1 |
| RZC07213 | BC1741 | 4.63 | 4.33 |  | malS | NAD-dependent malic enzyme | + |
| RZC04704# | BC1776 | 1.41 | 3.47 |  |  | Branched-chain amino acid aminotransferase | 1 |
| RZC03229# | BC1793 | 7.41 | 0.83 |  |  | Chlorohydrolase/deaminase family protein | 1 |
| RZC02616 | BC1809 | 2.28 | 1.92 |  | nheA | Non-hemolytic enterotoxin lytic component L2 | 2 |
| RZC02615 | BC1810 | 2.52 | 2.19 |  | nheB | Non-hemolytic enterotoxin lytic component L1 | + |
| RZC00546 | BC1811 | 1.80 | 2.26 |  | nheC | Non-expressed Enterotoxin C | + |
| RZC00128 | BC1821 | 4.40 | 3.19 |  | nupC | Nucleoside permease nupC | 1 |
| RZC00597 | BC1822 | 3.23 | 1.61 |  |  | Pyrimidine-nucleoside phosphorylase | + |
| RZC00598# | BC1823 | 4.47 | 2.81 |  |  | Cytidine deaminase | + |
| RZC10790 | BC1919 | 1.33 | 7.74 |  |  | DNA segregation ATPase FtsK/SpoIIIE and related proteins | - |
| RZC03656 | BC1958 | 3.79 | 1.61 |  | yoxD | 3-oxoacyl-[acyl-carrier protein] reductase | - |
| RZC05784 | BC1981 | 1.91 | 2.30 |  |  | Acyl carrier protein | - |
| RZC00506 | BC2018 | 1.97 | 2.12 |  | ycsN | IolS protein | 1 |
| RZC01590# | BC2052 | 1.17 | 2.64 |  |  | D-alanyl-D-alanine carboxypeptidase | - |
| RZC00121 | BC2120 | 1.60 | 2.32 |  | narJ | Respiratory nitrate reductase delta chain | - |
| RZC05828 | BC2130 | 0.95 | 3.02 |  |  | Transporter | - |
| RZC00361 | BC2178 | 1.86 | 2.69 |  |  | putative transcriptional regulator | 1 |
| RZC05773 | BC2179 | 4.14 | 4.21 |  | yhfO | Acetyltransferase | + |
| RZC01880 | BC2238 | 2.07 | 3.12 |  |  | Cereus group specific | 1 |
| RZC02493 | BC2300 | 1.01 | 2.60 |  |  | Oxalate/formate antiporter | 2 |
| RZC03804 | BC2332 | 1.68 | 2.62 |  |  | hypothetical protein | 1 |
| RZC03803 | BC2335 | 1.94 | 2.55 |  |  | Catabolite gene activator | - |
| RZC04614 | BC2403 | 1.47 | 2.54 |  |  | hypothetical protein | - |
| RZC04619 | BC2404 | 1.40 | 2.84 |  |  | hypothetical Cytosolic Protein | - |
| RZC04740 | BC2412 | 1.76 | 5.16 |  |  | ABC transporter permease protein | - |
| RZC04735 | BC2421 | 1.50 | 3.49 |  |  | DNA integration/recombination/invertion protein | - |
| RZC03776 | BC2519 | 1.75 | 4.32 |  |  | Lipase | - |
| RZC03630 | BC2543 | 1.30 | 2.87 |  | yxdL | ABC transporter ATP-binding protein | - |
| RZC03789 | BC2581 | 1.36 | 2.68 |  |  | Phage endonuclease | - |
| RZC05668 | BC2590 | 1.80 | 2.97 |  |  | Phage protein | - |
| RZC03239 | BC2691 | 1.76 | 3.26 |  |  | Acetyltransferase | - |
| RZC02190 | BC2766 | 1.32 | 1.21 | 3.18 | acoR | Sigma-54-dependent transcriptional activator | 1 |
| RZC01505 | BC2849 | 0.72 | 2.44 |  | ykfC | Cell wall-associated hydrolase | - |
| RZC07108 | BC2892 | 1.00 | 3.25 |  |  | hypothetical protein | 1 |
| RZC05863 | BC2925 | 0.97 | 4.04 |  |  | Nucleotidyltransferase | - |
| RZC01610 | BC2961 | 1.56 | 3.20 |  |  | Sugar transport system permease protein | 1 |
| RZC06472 | BC2962 | 1.61 | 2.87 |  |  | Sugar transport system permease protein | - |
| RZC02942 | BC2963 | 1.91 | 2.64 |  |  | Sugar transport ATP-binding protein | - |
| RZC05831 | BC2965 | 1.58 | 2.39 |  |  | Sugar (pentulose and hexulose) kinases | - |
| RZC03681 | BC2966 | 2.02 | 2.53 |  |  | Polyketide synthase curC | - |
| RZC10848 | BC3010 | 0.98 | 8.71 |  |  | Microbial collagenase | 1 |
| RZC01533 | BC3041 | 2.21 | 3.80 |  | ywsA | hypothetical protein | - |
| RZC01574 | BC3075 | 1.85 | 2.37 |  | ydbM | EpiH/GdmH-related protein | 1 |
| RZC06099 | BC3093 | 3.85 | 2.98 |  |  | Aspartate ammonia-lyase | - |
| RZC03946# | BC3189 | 1.21 | 2.07 |  |  | Serine transporter | 1 |
| RZC05986 | BC3193 | 2.45 | 3.26 |  |  | hypothetical protein | - |
| RZC10947 | BC3232 | 2.03 | 0.96 |  |  | hypothetical protein | - |
| RZC10939 | BC3234 | 2.07 | 0.75 |  |  | Glucose/mannose transporter | - |
| RZC03663 | BC3297 | 1.78 | 4.17 |  |  | hypothetical Cytosolic Protein | - |
| RZC02183 | BC3328 | 1.24 | 2.67 |  |  | ABC transporter permease protein | - |
| RZC07149 | BC3345 | 1.24 | 3.28 |  |  | Collagen-like triple helix repeat protein | - |
| RZC06014 | BC3446 | 2.29 | 1.59 |  |  | hypothetical protein | - |
| RZC03193 | BC3450 | 2.35 | 3.94 |  |  | hypothetical Membrane Spanning Protein | - |
| RZC03190 | BC3451 | 2.68 | 2.22 |  |  | hypothetical Membrane Spanning Protein | - |
| RZC03192 | BC3452 | 2.79 | 1.69 |  |  | hypothetical protein | - |
| RZC05715 | BC3477 | 1.80 | 2.72 |  |  | Quinone oxidoreductase | - |
| RZC05302 | BC3495 | 0.96 | 4.04 |  | glpQ | Glycerophosphoryl diester phosphodiesterase | - |
| RZC10859 | BC3547 | 1.88 | 7.33 |  |  | Cell surface protein | - |
| RZC02909 | BC3582 | 1.25 | 2.32 |  |  | hypothetical protein | - |
| RZC01093 | BC3585 | 2.53 | 1.20 |  |  | Oligopeptide-binding protein oppA | - |
| RZC04994 | BC3627 | 1.22 | 8.76 |  | yhfS | Acetyl-CoA acetyltransferase | - |
| RZC11028# | BC3649 | 3.69 | 1.42 |  | hutG | Formiminoglutamase (EC 3.5.3.8 | + |
| RZC00716# | BC3650 | 2.30 | 0.92 |  | hutI | Imidazolonepropionase | + |
| RZC00715# | BC3651 | 2.91 | 1.00 |  | hutU | Urocanate hydratase | + |
| RZC00321# | BC3652 | 2.44 | 0.96 |  | hutH | Histidine ammonia-lyase | 1 |
| RZC00429 | BC3681 | 1.73 | 2.50 |  | yneE | IG hypothetical 18106 | 1 |
| RZC00428 | BC3682 | 1.93 | 2.48 |  |  | Transketolase | - |
| RZC03035 | BC3788 | 5.94 | 3.86 |  | yufQ | Nucleoside transport system permease protein | 1 |
| RZC00501 | BC3789 | 7.18 | 9.34 |  | yufP | Nucleoside transport ATP-binding protein | 1 |
| RZC00502 | BC3790 | 2.16 | 3.65 |  | yufO | Nucleoside transport ATP-binding protein | 1 |
| RZC00041 | BC3791 | 8.88 | 9.88 |  | yufN | Nucleoside-binding protein | + |
| RZC00040 | BC3792 | 5.14 | 3.95 | 14.12 | ymfC | Transcriptional regulator, GntR family | 2 |
| RZC02281 | BC3833 | 7.74 | 7.57 |  | sucD | Succinyl-CoA synthetase alpha chain | + |
| RZC02282 | BC3834 | 8.23 | 6.32 |  | sucC | Succinyl-CoA synthetase beta chain | 1 |
| RZC02217 | BC3921 | 2.56 | 1.08 |  | ylbP | Acetyltransferase | 2 |
| RZC03103 | BC3985 | 1.30 | 2.14 |  | ykuK | hypothetical Cytosolic Protein | - |
| RZC02414# | BC4003 | 1.34 | 2.78 |  | metE | 5-methyltetrahydropteroyltriglutamate--homocysteine methyltransferase | - |
| RZC06591 | BC4023 | 2.34 | 0.93 |  |  | Acetyl-CoA acetyltransferase | - |
| RZC05779 | BC4083 | 3.15 | 1.47 |  |  | Guanine-hypoxanthine permease | 1 |
| RZC05592 | BC4086 | 2.18 | 0.72 |  | punA | Purine nucleoside phosphorylase | - |
| RZC01337 | BC4097 | 2.20 | 2.05 |  |  | 2,5-diketo-D-gluconic acid reductase | - |
| RZC00913# | BC4135 | 2.09 | 2.98 |  |  | L-serine dehydratase | + |
| RZC02465# | BC4136 | 2.24 | 2.62 |  |  | L-serine dehydratase | 1 |
| RZC01173# | BC4157 | 3.27 | 2.81 |  | bkdB | Lipoamide acyltransferase component of branched-chain alpha-keto acid dehydrogenase complex | + |
| RZC05713# | BC4158 | 3.84 | 2.14 |  |  | 2-oxoisovalerate dehydrogenase beta subunit | + |
| RZC01012# | BC4159 | 3.54 | 2.43 |  |  | 2-oxoisovalerate dehydrogenase alpha subunit | + |
| RZC01014# | BC4160 | 3.02 | 2.26 |  | lpdV | Dihydrolipoamide dehydrogenase | 1 |
| RZC02768# | BC4161 | 3.28 | 2.00 |  |  | Branched-chain-fatty-acid kinase | + |
| RZC02766# | BC4162 | 2.11 | 1.41 |  |  | Leucine dehydrogenase | 1 |
| RZC02767# | BC4163 | 2.23 | 1.51 |  |  | Phosphate butyryltransferase | 1 |
| RZC06622 | BC4200 | 1.61 | 3.88 |  |  | IG hypothetical 16750 | - |
| RZC01281 | BC4213 | 1.66 | 2.03 |  |  | Quaternary ammonium compound-resistance protein | - |
| RZC00112# | BC4224 | 2.30 | 1.65 |  |  | Glycine dehydrogenase [decarboxylating] | - |
| RZC01061# | BC4225 | 2.28 | 1.46 |  |  | Glycine dehydrogenase [decarboxylating] | - |
| RZC02167# | BC4226 | 2.43 | 1.31 |  | gcvT | Aminomethyltransferase | - |
| RZC00730 | BC4229 | 2.86 | 3.19 |  |  | hypothetical protein | 2 |
| RZC00050 | BC4362 | 2.05 | 1.38 |  |  | Ferrichrome transport system permease protein fhuB | - |
| RZC03701 | BC4510 | 2.58 | 1.90 |  | yhaQ | Sodium export ATP-binding protein | - |
| RZC07053 | BC4516 | 3.76 | 5.64 |  | sdhB | Succinate dehydrogenase iron-sulfur protein | - |
| RZC07054 | BC4517 | 4.42 | 3.70 |  | sdhA | Succinate dehydrogenase flavoprotein subunit | 1 |
| RZC05750 | BC4518 | 4.35 | 4.02 |  | sdhC | Succinate dehydrogenase cytochrome b558 subunit | 1 |
| RZC01079 | BC4523 | 3.06 | 0.85 |  | etfB | Electron transfer flavoprotein beta-subunit | - |
| RZC00195 | BC4524 | 2.48 | 0.83 |  | ysiB | 3-hydroxybutyryl-CoA dehydratase | - |
| RZC07229 | BC4583 | 1.74 | 2.79 | 4.26 | gapB | NAD(P)-dependent glyceraldehyde-3-phosphate dehydrogenase | 3 |
| RZC01264 | BC4592 | 5.81 | 4.32 |  |  | Malate dehydrogenase | + |
| RZC01263 | BC4593 | 5.56 | 4.38 |  |  | Isocitrate dehydrogenase [NADP] | 1 |
| RZC01265 | BC4594 | 3.76 | 2.23 |  | citZ | Citrate synthase | 1 |
| RZC06047 | BC4606 | 1.18 | 3.51 |  |  | hypothetical Membrane Spanning Protein | - |
| RZC02682 | BC4642 | 3.92 | 1.07 |  | ytdI | ATP-NAD kinase | 2 |
| RZC10893 | BC4825 | 1.57 | 4.44 |  |  | ABC transporter ATP-binding protein | - |
| RZC01614 | BC4961 | 3.33 | 2.02 |  | yutE | hypothetical Cytosolic Protein | 2 |
| RZC01716# | BC5006 | 2.38 | 0.95 |  | yusM | Prolyne dehydrogenase | 1 |
| RZC03472 | BC5009 | 2.29 | 2.06 |  | mcpC | Methyl-accepting chemotaxis protein | - |
| RZC03473 | BC5012 | 2.78 | 0.88 |  | ydhL | Chloramphenicol resistance protein | 1 |
| RZC08029# | BC5131 | 2.25 | 1.86 |  |  | Protein translocase subunit SecG | - |
| RZC02760 | BC5262 | 1.75 | 2.21 |  |  | Two-component response regulator | - |
| RZC04768 | BC5297 | 2.00 | 1.35 |  |  | NADH-quinone oxidoreductase chain H | - |
| RZC00783# | BC5319 | 2.24 | 2.24 |  | ywlE | Protein tyrosine phosphatase | - |
| RZC00125 | BC5333 | 3.73 | 3.34 |  | ywjI | Fructose-1,6-bisphosphatase | + |
| RZC08066 | BC5334 | 2.73 | 2.28 |  |  | UDP-N-acetylglucosamine 1-carboxyvinyltransferase | 1 |
| RZC08075 | BC5412 | 2.16 | 1.84 |  |  | Two-component sensor protein yhcY | - |
| RZC01109 | BC5439 | 2.11 | 6.54 |  | ysbA | Murein hydrolase exporter | 1 |
| RZC02320 | BC5447 | 2.44 | 0.87 |  |  | Acetamide transporter | 1 |
|  |  |  |  |  |  |  |  |
| RZC06834 | BC0189 | 0.45 | 0.43 |  |  | hypothetical protein | 1 |
| RZC04620 | BC0190 | 0.48 | 0.66 |  | glmS | Glucosamine--fructose-6-phosphate aminotransferase [isomerizing] | + |
| RZC04626 | BC0194 | 0.45 | 0.63 |  |  | hypothetical Membrane Spanning Protein | - |
| RZC03294 | BC0219 | 0.22 | 0.13 |  |  | Glucose uptake protein homolog | 2 |
| RZC05193 | BC0228 | 0.49 | 0.81 |  | yhdO | 1-acyl-sn-glycerol-3-phosphate acyltransferase | - |
| RZC03560 | BC0253 | 0.50 | 0.56 |  |  | Fumarylacetoacetase | - |
| RZC05027 | BC0360 | 0.69 | 0.44 |  | ampS | Aminopeptidase | - |
| RZC02100 | BC0595 | 0.78 | 0.45 |  | yozA | Cadmium efflux system accessory protein | - |
| RZC01585 | BC0612 | 0.34 | 1.21 |  |  | L-lactate permease | - |
| RZC07860 | BC0647 | 0.86 | 0.48 |  |  | RNA polymerase ECF-type sigma factor | - |
| RZC03830 | BC0655 | 0.50 | 0.85 |  |  | Universal stress protein family | - |
| RZC05711 | BC0683 | 0.96 | 0.47 |  |  | hypothetical protein | - |
| RZC06313 | BC0685 | 0.49 | 0.50 |  |  | Branched-chain amino acid transport system carrier protein | - |
| RZC00046 | BC0694 | 0.43 | 0.33 |  | yjbQ | Na+/H+ antiporter NapA | - |
| RZC03841 | BC0868 | 0.31 | 0.26 |  | gapN | NADP-dependent glyceraldehyde-3-phosphate dehydrogenase | 2 |
| RZC07169 | BC0907 | 0.70 | 0.43 |  | appA | Oligopeptide-binding protein oppA | 1 |
| RZC06880 | BC1060 | 0.30 | 0.67 |  |  | Collagen adhesion protein | - |
| RZC03622 | BC1181 | 2.07 | 0.50 |  | oppC | Oligopeptide transport system permease protein oppC | - |
| RZC05965 | BC1183 | 1.79 | 0.48 |  | oppF | Oligopeptide transport ATP-binding protein oppF | - |
| RZC00487 | BC1435 | 0.37 | 0.76 |  |  | hypothetical protein | - |
| RZC03432 | BC1908 | 0.54 | 0.36 |  |  | hypothetical protein | - |
| RZC05932 | BC1924 | 0.44 | 1.05 |  |  | L-lactate dehydrogenase | - |
| RZC00144 | BC2035 | 0.38 | 0.74 |  |  | Magnesium and cobalt transport protein corA | 2 |
| RZC00219 | BC2121 | 0.53 | 0.49 |  | narI | Respiratory nitrate reductase gamma chain | - |
| RZC01018 | BC2369 | 0.47 | 0.46 |  |  | Acetyltransferase | - |
| RZC08307 | BC2453 | 0.55 | 0.44 |  | ppsC | Peptide synthetase | - |
| RZC00208 | BC2771 | 0.36 | 0.58 |  |  | hypothetical protein | - |
| RZC00218 | BC2795 | 0.29 | 0.35 |  |  | Ferredoxin | - |
| RZC05794 | BC3221 | 0.46 | 0.87 |  |  | surface protein | - |
| RZC02185 | BC3329 | 0.91 | 0.31 |  |  | ABC transporter ATP-binding protein | - |
| RZC06783 | BC3557 | 1.49 | 0.24 |  | sqhC | Squalene—hopene cyclase | - |
| RZC06776 | BC3595 | 0.66 | 0.45 |  | yvaA | Oxidoreductase | 1 |
| RZC01178 | BC3720 | 0.51 | 0.73 | 0.79 | fruR | Fructose repressor | 1 |
| RZC00386 | BC3848 | 0.39 | 1.02 |  | acpA | Acyl carrier protein | - |
| RZC06588 | BC3857 | 1.17 | 0.12 |  | yloS | Thiamin pyrophosphokinase | - |
| RZC06584 | BC3877 | 1.32 | 0.44 |  |  | hypothetical Membrane Spanning Protein | - |
| RZC06581 | BC3880 | 1.23 | 0.48 |  |  | hypothetical Membrane Spanning Protein | 2 |
| RZC02045 | BC3883 | 0.83 | 0.19 |  | pyrF | Orotidine 5'-phosphate decarboxylase | - |
| RZC02044 | BC3884 | 0.88 | 0.20 |  | pyrD | Dihydroorotate dehydrogenase, catalytic subunit | - |
| RZC06579 | BC3885 | 1.02 | 0.14 |  | pyrK | Dihydroorotate dehydrogenase electron transfer subunit | - |
| RZC06578 | BC3886 | 1.04 | 0.11 |  |  | Carbamoyl-phosphate synthase large chain | - |
| RZC02365 | BC3888 | 1.14 | 0.33 |  | pyrC | Dihydroorotase | - |
| RZC05814 | BC3889 | 0.75 | 0.30 |  | pyrB | Aspartate carbamoyltransferase | - |
| RZC05813 | BC3890 | 0.69 | 0.25 |  | pyrP | Uracil permease | - |
| RZC00634 | BC3931 | 0.42 | 1.06 |  |  | hypothetical protein | - |
| RZC01068 | BC3996 | 0.18 | 0.58 |  |  | hypothetical protein | - |
| RZC02890 | BC4017 | 0.32 | 0.30 |  |  | 3-oxoacyl-[acyl-carrier protein] reductase | 1 |
| RZC05706 | BC4088 | 0.45 | 1.09 |  | ytkA | IG hypothetical 17224 | - |
| RZC00154 | BC4240 | 0.27 | 1.02 |  |  | Transcriptional regulator | 1 |
| RZC03283 | BC4366 | 0.74 | 0.45 |  | yrhB | Cystathionine beta-lyase | - |
| RZC01414 | BC4599 | 0.29 | 0.33 |  |  | Pyruvate kinase | - |
| RZC02283 | BC4600 | 0.45 | 0.47 |  | pfkA | 6-phosphofructokinase | - |
| RZC00434 | BC4658 | 0.29 | 0.60 |  |  | Maltose O-acetyltransferase | - |
| RZC04869 | BC4669 | 0.47 | 0.54 |  | mscL | Large-conductance mechanosensitive channel | 1 |
| RZC03693 | BC4766 | 0.30 | 0.26 |  |  | hypothetical Cytosolic Protein | - |
| RZC03793 | BC4898 | 0.31 | 0.20 |  |  | Glucose-6-phosphate isomerase | 1 |
| RZC05291 | BC5018 | 0.46 | 0.58 |  |  | hypothetical Membrane Spanning Protein | 1 |
| RZC05299 | BC5026 | 0.50 | 1.11 |  |  | hypothetical protein | - |
| RZC06290 | BC5051 | 0.93 | 0.41 |  | alsT | Sodium/proton-dependent alanine carrier protein | - |
| RZC05504 | BC5116 | 0.44 | 0.81 |  |  | hypothetical protein | - |
| RZC05498 | BC5117 | 0.67 | 0.47 |  |  | ABC transporter permease protein | - |
| RZC05499 | BC5118 | 0.76 | 0.48 |  |  | ABC transporter ATP-binding protein | - |
| RZC05500 | BC5119 | 0.68 | 0.45 |  |  | hypothetical protein | - |
| RZC05497 | BC5121 | 0.67 | 0.48 |  |  | hypothetical protein | - |
| RZC05494 | BC5122 | 0.68 | 0.49 |  |  | hypothetical Cytosolic Protein | - |
| RZC05501 | BC5123 | 0.69 | 0.48 |  |  | hypothetical protein | 1 |
| RZC05490 | BC5124 | 0.66 | 0.48 |  |  | hypothetical protein | - |
| RZC05496 | BC5125 | 0.68 | 0.42 |  |  | hypothetical protein | - |
| RZC02971 | BC5135 | 0.42 | 0.51 |  |  | Enolase | - |
| RZC05843 | BC5136 | 0.48 | 0.38 |  |  | Phosphoglycerate mutase | - |
| RZC07001 | BC5137 | 0.50 | 0.41 |  | tpiA | Triosephosphate isomerase (EC 5.3.1.1) | - |
| RZC08031 | BC5138 | 0.53 | 0.46 |  |  | Phosphoglycerate kinase [fragment] (EC 2.7.2.3) | - |
| RZC00211 | BC5139 | 0.49 | 0.41 |  |  | Phosphoglycerate kinase [fragment] (EC 2.7.2.3) | - |
| RZC00210 | BC5140 | 0.36 | 0.59 |  | gapA | Glyceraldehyde 3-phosphate dehydrogenase (EC 1.2.1.12) | - |
| RZC08032 | BC5141 | 0.49 | 0.73 | 0.33 | cggR | Central glycolytic genes regulator | - |
| RZC00782 | BC5320 | 0.32 | 0.24 |  | ypqE | PTS system, glucose-specific IIA component | 1 |
| RZC03557 | BC5335 | 0.39 | 0.52 |  | fbaA | Fructose-bisphosphate aldolase | 1 |
| RZC05846 | BC5359 | 0.45 | 0.47 |  | ywaD | Aminopeptidase Y | - |
| RZC01390 | BC5368 | 0.38 | 0.27 |  |  | Transcriptional regulator pfoR | - |
| RZC05205 | BC5380 | 0.46 | 0.52 |  |  | Ferrichrome-binding protein | - |
| RZC04933 | BC5396 | 0.49 | 0.44 |  | yxeH | hydrolase (HAD superfamily) | 1 |

* Expression ratio’s are presented as in the *ccpA* deletion strain compared to the wild type, wild type expression is set to 1. Early-exponential is sampled at an OD_600_ of 0.2 and mid-exponential at 0.8.

† Expression ratio’s of qPCR are presented as in the ccpA deletion strain compared to the wild type, gene expression is related to the household gene *rpoA*.

‡ In this column it is indicated whether a putative CRE-site was identified for the gene. Numbers indicate the number of putative CRE-sites identified, a + indicates that a gene is in the same operon as a gene for which a putative CRE-site was identified, and a – indicates no putative CRE-site could be identified.

§ Possibly in operon with gene for which a CRE-site was identified, however this gene was absent from the microarray.

# Genes, higher expressed in the *ccpA* deletion strain, with an apparent putative function in protein, peptide or amino acid metabolism.
